# Visceral Adiposity Is Independently Associated with Hypertension in Thai Adults: A Case–Control Study

**DOI:** 10.3390/ijerph23081057

**Published:** 2026-08-14

**Authors:** Pattaraphorn Srikaew, Surachat Buddhisa, Surada Satthakarn, Nattaphol Prakobkaew, Tadsawiya Padkao, Orachorn Boonla, Piyapong Prasertsri

**Affiliations:** Faculty of Allied Health Sciences, Burapha University, Chonburi 20131, Thailand; 68910057@go.buu.ac.th (P.S.); surachat.bu@go.buu.ac.th (S.B.); surada.sa@go.buu.ac.th (S.S.); nattaphol@go.buu.ac.th (N.P.); tadsawiya@go.buu.ac.th (T.P.); orachorn@go.buu.ac.th (O.B.)

**Keywords:** hypertension, body composition, visceral adiposity, visceral fat, body fat percentage, skeletal muscle mass, cardiovascular risk, Thai adults

## Abstract

**Highlights:**

**Public health relevance—How does this work relate to a public health issue?**

- Hypertension remains a major public health challenge worldwide, underscoring the need to identify modifiable body composition characteristics associated with elevated blood pressure.
- Visceral adiposity may provide more clinically relevant information for hypertension risk assessment than conventional anthropometric measures alone.

**Public health significance—Why is this work of significance to public health?**

- Thai adults with hypertension exhibited significantly greater overall and regional adiposity, including higher body fat percentage, fat mass, visceral fat level, and segmental fat mass, than normotensive individuals.
- Visceral fat level remained independently associated with hypertension after adjustment for age, sex, and skeletal muscle mass, whereas skeletal muscle mass showed no independent association.

**Public health implications—What are the key implications or messages for practitioners, policy makers and/or researchers in public health?**

- Assessment of visceral adiposity may complement conventional anthropometric screening to improve identification of Thai adults at increased risk of hypertension.
- Public health strategies targeting reduction in visceral fat through healthy diet, regular physical activity, and weight management may contribute to hypertension prevention and improved cardiovascular health.

**Abstract:**

Background: Hypertension is a major public health challenge and a leading contributor to cardiovascular morbidity and mortality worldwide. Although obesity is a well-established risk factor for hypertension, the body composition characteristics most strongly associated with hypertension remain unclear. This study aimed to investigate the associations between body composition parameters and hypertension among Thai adults. Methods: This case–control study included 208 Thai adults aged ≥18 years, comprising 103 normotensive individuals and 105 individuals with hypertension. Blood pressure indices included systolic blood pressure (SBP), diastolic blood pressure (DBP), pulse pressure (PP), mean arterial pressure (MAP), and rate-pressure product (RPP). Body composition was assessed using bioelectrical impedance analysis (BIA) and included overall and regional body composition parameters, including visceral fat level, fat mass, body fat percentage, and skeletal muscle mass. Differences between groups were examined, and multivariable logistic regression analyses were performed to identify factors independently associated with hypertension. Results: Compared with normotensive participants, individuals with hypertension were significantly older (*p* < 0.001) and more likely to be male (*p* = 0.010). They also exhibited significantly greater adiposity-related characteristics, including higher body weight (*p* = 0.001), body mass index (*p* = 0.001), body fat percentage (*p* = 0.023), fat mass (*p* < 0.001), visceral fat level (*p* < 0.001), and waist-to-hip ratio (*p* = 0.037), together with higher SBP, DBP, PP, MAP, and RPP (all *p* < 0.001). In Model 3, older age (OR = 1.06, 95% CI: 1.04–1.09; *p* < 0.001), male sex (OR = 5.35, 95% CI: 1.69–16.95; *p* = 0.004), and higher visceral fat level (OR = 1.16, 95% CI: 1.06–1.26; *p* < 0.001) were associated with hypertension, whereas skeletal muscle mass was not (OR = 1.03, 95% CI: 0.95–1.12; *p* = 0.438). Each one-unit increase in visceral fat level was associated with a 16% increase in the odds of hypertension. The final model demonstrated acceptable discrimination (AUC = 0.79, 95% CI: 0.72–0.85). Conclusions: A higher BIA-derived visceral fat level was associated with hypertension among Thai adults after adjustment for age, sex, and skeletal muscle mass, whereas skeletal muscle mass showed no independent association in the same model. These findings suggest that visceral fat distribution may provide complementary information in cardiometabolic assessment. However, prospective studies using imaging-based assessments are required to confirm these findings.

## 1. Introduction

Hypertension is one of the most prevalent non-communicable diseases worldwide and remains a leading contributor to cardiovascular morbidity and mortality [1]. Elevated blood pressure substantially increases the risk of coronary artery disease, stroke, heart failure, chronic kidney disease, and premature death [2]. Despite advances in prevention and treatment, the global burden of hypertension continues to rise, particularly in low- and middle-income countries [3]. In Thailand, hypertension represents a major public health challenge and is an important contributor to healthcare expenditures and cardiovascular disease burden [4]. Therefore, identifying modifiable factors associated with hypertension remains a public health priority.

Obesity is a well-established risk factor for hypertension and contributes to BP elevation through multiple mechanisms, including activation of the sympathetic nervous system and the renin–angiotensin–aldosterone system, endothelial dysfunction, insulin resistance, and chronic low-grade inflammation [5,6]. Traditionally, obesity-related cardiovascular risk has been assessed using anthropometric indices such as body mass index (BMI), waist circumference, and waist-to-hip ratio [7]. However, these measures provide limited information regarding body composition because they do not distinguish between fat mass and lean mass or characterize fat distribution. Consequently, individuals with similar BMI values may have substantially different cardiometabolic risk profiles.

Emerging evidence suggests that visceral adiposity is an important marker of cardiometabolic risk and may provide additional information beyond conventional anthropometric measures [8,9,10,11,12]. This issue may be particularly relevant in Asian populations, who tend to develop cardiometabolic abnormalities at lower BMI levels and exhibit greater visceral adiposity than Western populations with comparable BMI values [13]. Therefore, assessment of visceral adiposity may provide additional information for hypertension risk stratification.

In contrast, the role of skeletal muscle mass in hypertension remains less clear. Skeletal muscle is an important metabolic tissue involved in glucose utilization, energy expenditure, and vascular health [14]. Several studies have suggested that reduced muscle mass may be associated with adverse cardiometabolic outcomes [15,16]; however, findings regarding its independent relationship with hypertension have been inconsistent. It remains uncertain whether skeletal muscle mass contributes independently to hypertension after accounting for adiposity-related factors. In addition, evidence remains inconsistent regarding whether overall and regional body composition characteristics contribute independently to hypertension beyond established risk factors such as age, sex, and overall adiposity.

To date, evidence regarding the independent contributions of visceral adiposity and skeletal muscle mass to hypertension remains limited, particularly in Southeast Asian populations. Furthermore, few studies have comprehensively evaluated overall and regional body composition characteristics in Thai adults. Therefore, this study aimed to investigate the associations between overall and regional body composition parameters and hypertension among Thai adults and to identify the body composition parameters independently associated with hypertension after adjustment for relevant covariates and potential confounders.

## 2. Materials and Methods

### 2.1. Study Design and Sample Size

This case–control study was conducted among Thai adults with hypertension and normotensive controls. The required sample size was estimated based on the correlation coefficient reported by Phababpha et al. [17] using the sample size calculator available at http://powerandsamplesize.com/Calculators/ (accessed on 10 October 2023). Assuming an expected correlation coefficient of 0.20, a two-sided significance level (α) of 0.05, and a statistical power of 80%, the minimum required sample size was calculated to be 194 participants. To account for potential missing or incomplete data and to ensure adequate statistical power, the target sample size was increased to at least 200 participants.

### 2.2. Ethical Considerations and Informed Consent

This study was conducted in accordance with the ethical principles of the Declaration of Helsinki and was approved by the Human Research Ethics Committee of Burapha University (Approval No. IRB1-046/2568; approval date: 29 April 2025). Written informed consent was obtained from all participants before screening and data collection. The study was prospectively registered at ClinicalTrials.gov (Identifier: NCT07018128; registration date: 1 June 2025).

### 2.3. Recruitment and Screening of Participants

Participants were recruited and assessed between June 2025 and March 2026 through advertisements posted at community health centers, hospitals, municipal offices, and social media platforms (Facebook and LINE). Individuals who expressed interest in participating were provided with a brief explanation of the study objectives and procedures and were subsequently invited to attend an in-person screening visit. During this visit, trained research assistants provided a detailed explanation of the study, obtained medical history, and measured body weight, height, and BMI to assess eligibility. Individuals who met the eligibility criteria and agreed to participate provided written informed consent before enrollment and were scheduled for study assessments.

Eligible participants were adults aged ≥18 years who were classified as either normotensive or hypertensive. Hypertension was defined as systolic blood pressure (SBP) ≥140 mmHg and/or diastolic blood pressure (DBP) ≥ 90 mmHg [18], or a previous physician diagnosis of hypertension with current antihypertensive treatment, irrespective of measured BP at study enrollment. The hypertension group included both treated individuals with established hypertension and newly identified cases meeting the study diagnostic criteria. The normotensive group comprised individuals without a previous diagnosis of hypertension whose BP values were below the diagnostic thresholds. All participants were required to have permanent residency in Thailand and to be living in the Eastern region at the time of enrollment.

Individuals were excluded if they had a history of cardiovascular disease or were experiencing an acute febrile or infectious illness, including coronavirus disease 2019 (COVID-19), influenza, or other infectious diseases, at the time of enrollment.

### 2.4. Data Collection Procedures

#### 2.4.1. Participant Preparation

Following the screening visit, eligible participants were scheduled to attend the research laboratory at approximately 8:00 a.m. for data collection. Prior to the visit, participants were instructed to: (1) obtain at least 7 h of sleep on the night before the assessment; (2) fast for at least 8 h, with only small amounts of water permitted if necessary; (3) refrain from consuming tea, coffee, alcohol, and tobacco products for at least 4 h before the assessment; and (4) avoid moderate- to vigorous-intensity physical activity, including structured exercise and strenuous household activities, during the 24 h preceding the visit.

#### 2.4.2. Participant Measurements

Upon arrival at the research laboratory, participants rested in a seated position for 15 min before all measurements were obtained. BP indices, including SBP and DBP, together with heart rate (HR), were measured using a validated automated BP monitor. Peripheral oxygen saturation (SpO_2_) was assessed using a vital signs and cardiac monitoring device. Pulse pressure (PP), mean arterial pressure (MAP), and rate-pressure product (RPP) were subsequently calculated from the measured blood pressure and heart rate values.

Body composition was then assessed using a bioelectrical impedance analyzer. The measured variables included body weight, BMI, body fat percentage, fat mass, total body water, skeletal muscle mass, protein mass, mineral mass, visceral fat level, waist-to-hip ratio, basal metabolic rate (BMR), and fitness score.

### 2.5. Blood Pressure Measurement

Following a 15 min rest in a seated position, BP was measured three consecutive times at 2 min intervals using a validated automated BP monitor (Omron HEM-705CP; Omron Healthcare, Kyoto, Japan) with a measurement accuracy of ±3 mmHg. An appropriately sized cuff was placed on the upper arm, with the lower edge positioned approximately 2.5 cm (1 inch) above the antecubital fossa.

SBP, DBP, and HR were recorded at each measurement. The average of the two closest BP readings was used for the final analysis. PP was calculated as SBP − DBP, MAP as DBP + (PP/3), and RPP as HR × SBP.

### 2.6. Anthropometric Assessment

Anthropometric and body composition measurements were performed using a multifrequency bioelectrical impedance analyzer (InBody270, InBody Co., Ltd., Daejeon, Republic of Korea). Participants were assessed in the standing position while wearing light clothing and no shoes. During the measurement, participants stood upright with their feet positioned on the foot electrodes and their arms slightly abducted while holding the hand electrodes, following the manufacturer’s instructions. Participant information, including sex, age, and height, was entered into the device before each assessment. The measurement was completed within approximately 1 min.

To improve measurement accuracy, participants were instructed to void their bladder immediately before the assessment. They were also advised to empty their bowels whenever possible and to avoid alcohol consumption, caffeine-containing beverages, smoking, and vigorous physical activity for at least 24 h before the measurement.

### 2.7. Statistical Analysis

All statistical analyses were performed using IBM SPSS Statistics for Windows, version 25.0 (IBM Corp., Armonk, NY, USA). All statistical tests were two-sided, and a *p* value < 0.05 was considered statistically significant. Data normality was assessed using the Shapiro–Wilk test, together with visual inspection of histograms and normal Q–Q plots. Continuous variables are presented as mean ± standard deviation (SD), whereas categorical variables are presented as frequencies and percentages.

Between-group comparisons were performed using the independent-samples *t*-test for normally distributed continuous variables and the Mann–Whitney U test for non-normally distributed variables. Categorical variables were compared using the chi-square (χ^2^) test. Cohen’s *d* was calculated for continuous variables, whereas Cohen’s *h* was calculated for differences in proportions. Absolute effect-size values of approximately 0.20, 0.50, and 0.80 were interpreted as small, medium, and large, respectively.

To identify factors associated with hypertension, univariate logistic regression analyses were initially performed for selected demographic and body composition variables. Variables with *p* < 0.10 in the univariate analyses or considered clinically relevant based on previous literature were subsequently entered into multivariable logistic regression models to identify body composition parameters independently associated with hypertension. Odds ratios (ORs) and 95% confidence intervals (CIs) were calculated. Three multivariable models were constructed to evaluate the independent contributions of body composition parameters to hypertension. Specifically, Model 1 included age, sex, and BMI; Model 2 included age, sex, body fat percentage, and visceral fat level; and Model 3 included age, sex, visceral fat level, and skeletal muscle mass.

Model performance was evaluated using the Hosmer–Lemeshow goodness-of-fit test, Nagelkerke’s R^2^, and the area under the receiver operating characteristic (ROC) curve (AUC). Multicollinearity among predictor variables was assessed using the variance inflation factor (VIF), with VIF values < 5 considered indicative of the absence of problematic multicollinearity.

## 3. Results

### 3.1. Participant Characteristics

**Figure 1:**
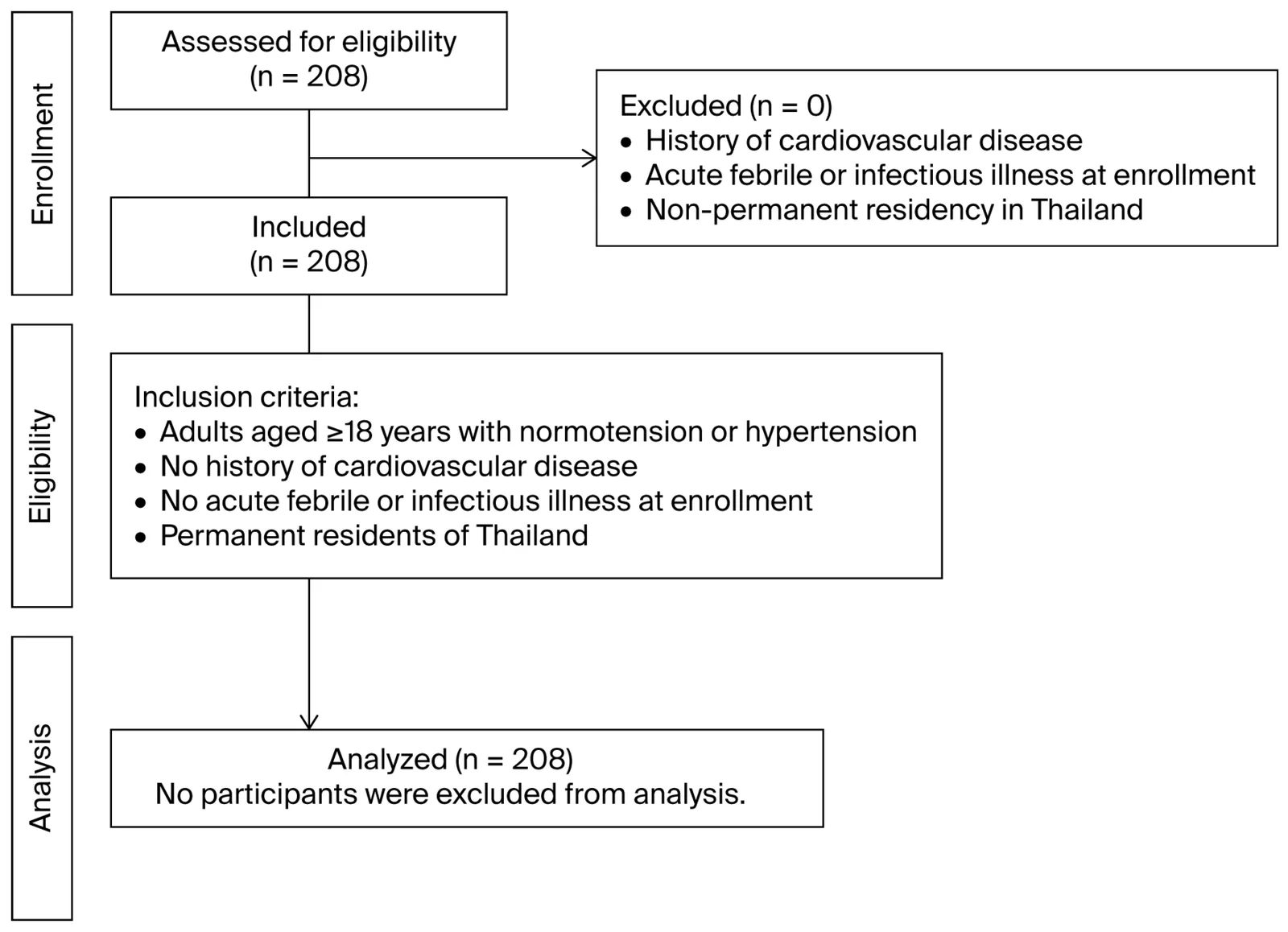
STROBE flow diagram illustrating participant recruitment, eligibility assessment, enrollment, and inclusion in the final analysis.

A total of 208 Thai adults who met the inclusion and exclusion criteria were enrolled in the study, and all participants were included in the final analyses (Figure 1).

Of the 208 participants, 161 (77.4%) were female, and 47 (22.6%) were male. Based on BP classification, 103 participants (49.5%) were normotensive and 105 (50.5%) had hypertension. Among participants with hypertension, 46 (43.81%) were receiving antihypertensive treatment, whereas 59 (56.19%) were newly diagnosed at study enrollment. Clinical and lifestyle characteristics, including antihypertensive treatment status, use of lipid-lowering medications, smoking status, alcohol consumption, and regular exercise participation, are summarized in Table 1.

#### 3.1.1. Demographic, Clinical, Anthropometric, Body Composition, and Hemodynamic Characteristics of Participants

**Figure 2:**
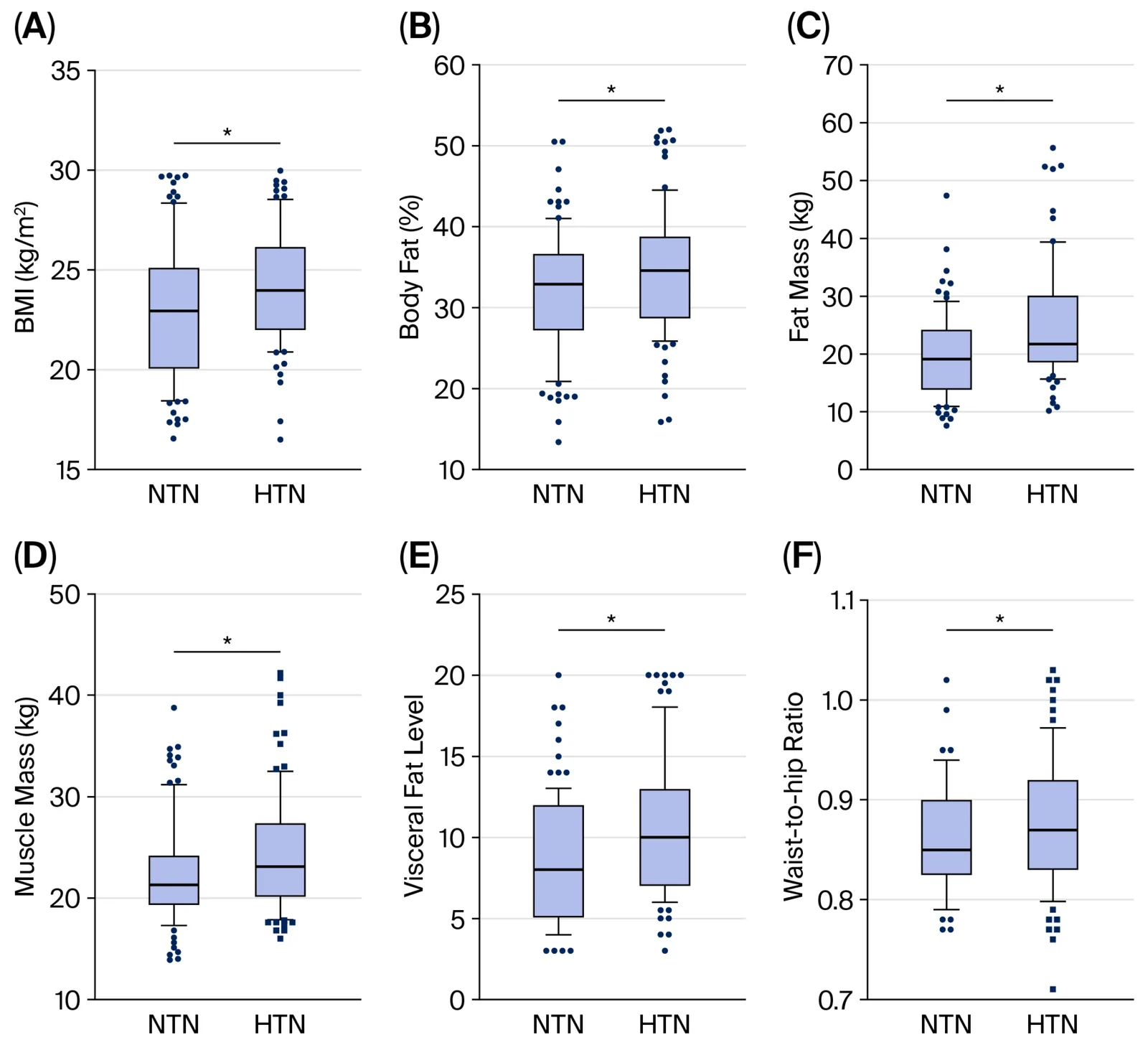
Box-and-whisker plots of key body composition parameters, including body mass index (BMI) (**A**), body fat percentage (**B**), fat mass (**C**), skeletal muscle mass (**D**), visceral fat level (**E**), and waist-to-hip ratio (**F**), in normotensive (NTN) and hypertensive (HTN) participants. Boxes represent the interquartile range (25th–75th percentiles), horizontal lines within the boxes indicate the medians, whiskers indicate the 10th and 90th percentiles, and points outside the whiskers represent observations below the 10th or above the 90th percentile. * *p* < 0.05 between groups.

The physical, physiological, and clinical characteristics of the participants are presented in Table 1. Compared with normotensive participants, individuals with hypertension were significantly older (*p* < 0.001) and more likely to be male (*p* = 0.010). They also exhibited significantly greater adiposity-related characteristics, including higher body weight (*p* = 0.001), BMI (*p* = 0.001), body fat percentage (*p* = 0.023), fat mass (*p* < 0.001), visceral fat level (*p* < 0.001), and waist-to-hip ratio (*p* = 0.037). In addition, participants with hypertension had significantly higher skeletal muscle mass (*p* = 0.027), protein mass (*p* = 0.047), mineral mass (*p* = 0.044), and BMR (*p* = 0.042), as well as higher SBP, DBP, PP, MAP, and RPP (all *p* < 0.001), whereas fitness score was significantly lower (*p* = 0.007) (Figure 2). No significant between-group differences were observed for height, water mass, HR, or SpO_2_ (all *p* > 0.05). Most between-group differences in body composition parameters were of small-to-moderate magnitude, whereas BP-related indices demonstrated large effect sizes.

#### 3.1.2. Segmental Muscle and Fat Mass of Participants

**Figure 3:**
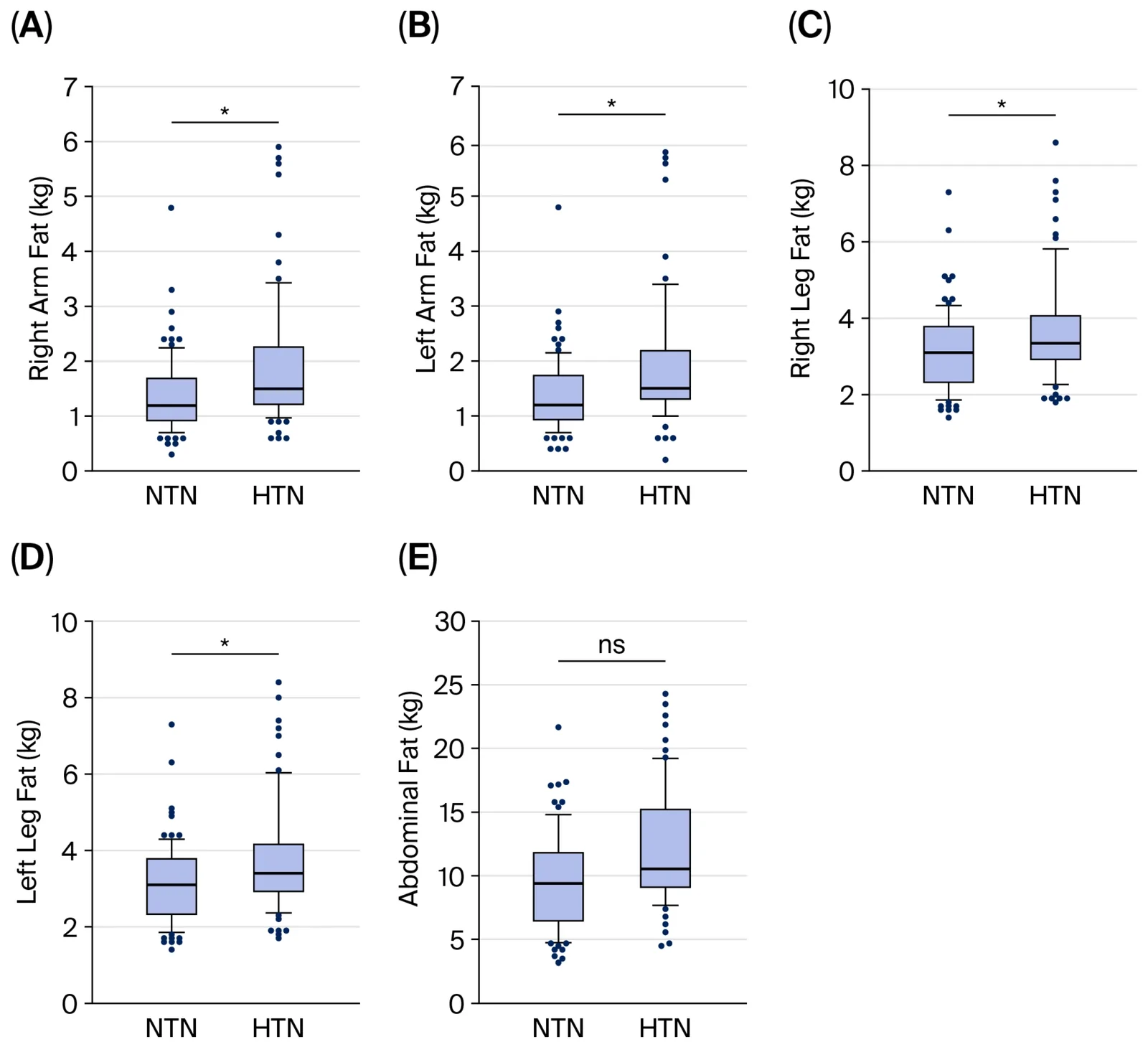
Box-and-whisker plots of segmental fat mass, including right arm fat (**A**), left arm fat (**B**), right leg fat (**C**), left leg fat (**D**), and abdominal fat (**E**), in normotensive (NTN) and hypertensive (HTN) participants. Boxes represent the interquartile range (25th–75th percentiles), horizontal lines within the boxes indicate the medians, whiskers indicate the 10th and 90th percentiles, and points outside the whiskers represent observations below the 10th or above the 90th percentile. * *p* < 0.05 between groups; ns, not significant.

Segmental body composition data are presented in Table 2. Compared with normotensive participants, individuals with hypertension exhibited significantly greater muscle mass in both upper extremities and the left leg (*p* = 0.028–0.043), whereas no significant differences were observed for right leg or abdominal muscle mass (both *p* > 0.05). Fat mass was also significantly greater in both upper and lower extremities (*p* < 0.001–0.003), while abdominal fat mass did not differ significantly between groups (*p* = 0.164) (Figure 3).

### 3.2. Associations Between Key Body Composition Indices and Hypertension

To identify key body composition characteristics associated with hypertension, selected demographic and body composition variables were examined using univariate logistic regression analyses. Older age (OR = 1.04, 95% CI: 1.02–1.05, *p* < 0.001), male sex (OR = 2.57, 95% CI: 1.29–5.11, *p* = 0.007), higher BMI (OR = 1.10, 95% CI: 1.04–1.17, *p* = 0.002), body fat percentage (OR = 1.04, 95% CI: 1.01–1.08, *p* = 0.025), fat mass (OR = 1.08, 95% CI: 1.03–1.12, *p* = 0.001), visceral fat level (OR = 1.15, 95% CI: 1.07–1.23, *p* < 0.001), and skeletal muscle mass (OR = 1.06, 95% CI: 1.01–1.12, *p* = 0.030) were each significantly associated with hypertension in the univariate analyses (Table 3).

### 3.3. Independent Associations Between Key Body Composition Indices and Hypertension

**Figure 4:**
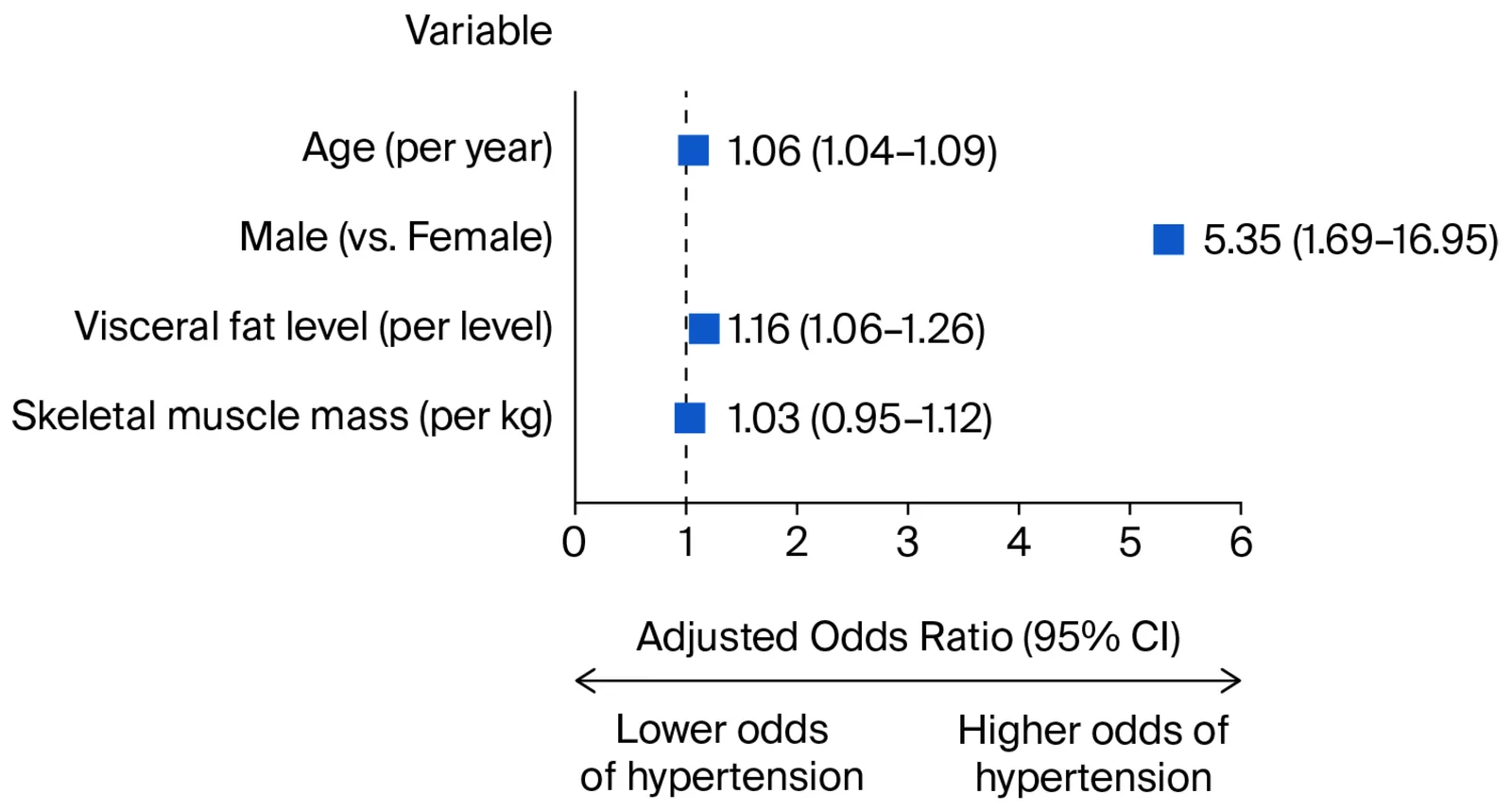
Forest plot of Model 3 from the multivariable logistic regression analysis showing factors independently associated with hypertension. Odds ratios (ORs) and 95% confidence intervals (CIs) are shown. The dotted vertical line indicates the null value (OR = 1.0).

Multivariable logistic regression analyses are presented in Table 4. In Model 1, older age (OR = 1.06, 95% CI: 1.04–1.08, *p* < 0.001), male sex (OR = 5.95, 95% CI: 2.44–14.51, *p* < 0.001), and higher BMI (OR = 1.11, 95% CI: 1.04–1.19, *p* = 0.002) were independently associated with hypertension. In Model 2, neither body fat percentage (OR = 1.00, 95% CI: 0.91–1.09, *p* = 0.950) nor visceral fat level (OR = 1.17, 95% CI: 1.00–1.37, *p* = 0.055) showed an independent association with hypertension after adjustment for age and sex. In Model 3, older age (OR = 1.06, 95% CI: 1.04–1.09, *p* < 0.001), male sex (OR = 5.35, 95% CI: 1.69–16.95, *p* = 0.004), and higher visceral fat level (OR = 1.16, 95% CI: 1.06–1.26, *p* < 0.001) remained independently associated with hypertension, whereas skeletal muscle mass was not (OR = 1.03, 95% CI: 0.95–1.12, *p* = 0.438) (Figure 4). Notably, visceral fat level was independently associated with hypertension only in Model 3 after adjustment for age, sex, and skeletal muscle mass.

#### Model Performance of Multivariable Logistic Regression Analysis for Hypertension

All three models demonstrated acceptable discrimination, with AUC values ranging from 0.76 to 0.79 (Table 5). Model 3 had the numerically highest AUC; however, differences between models were interpreted descriptively because formal pairwise comparisons of AUCs were not performed. Nagelkerke’s R^2^ values ranged from 0.28 to 0.37. No problematic multicollinearity was identified, with maximum VIF values ranging from 1.32 to 2.11.

### 3.4. ROC Curve Analysis

**Figure 5:**
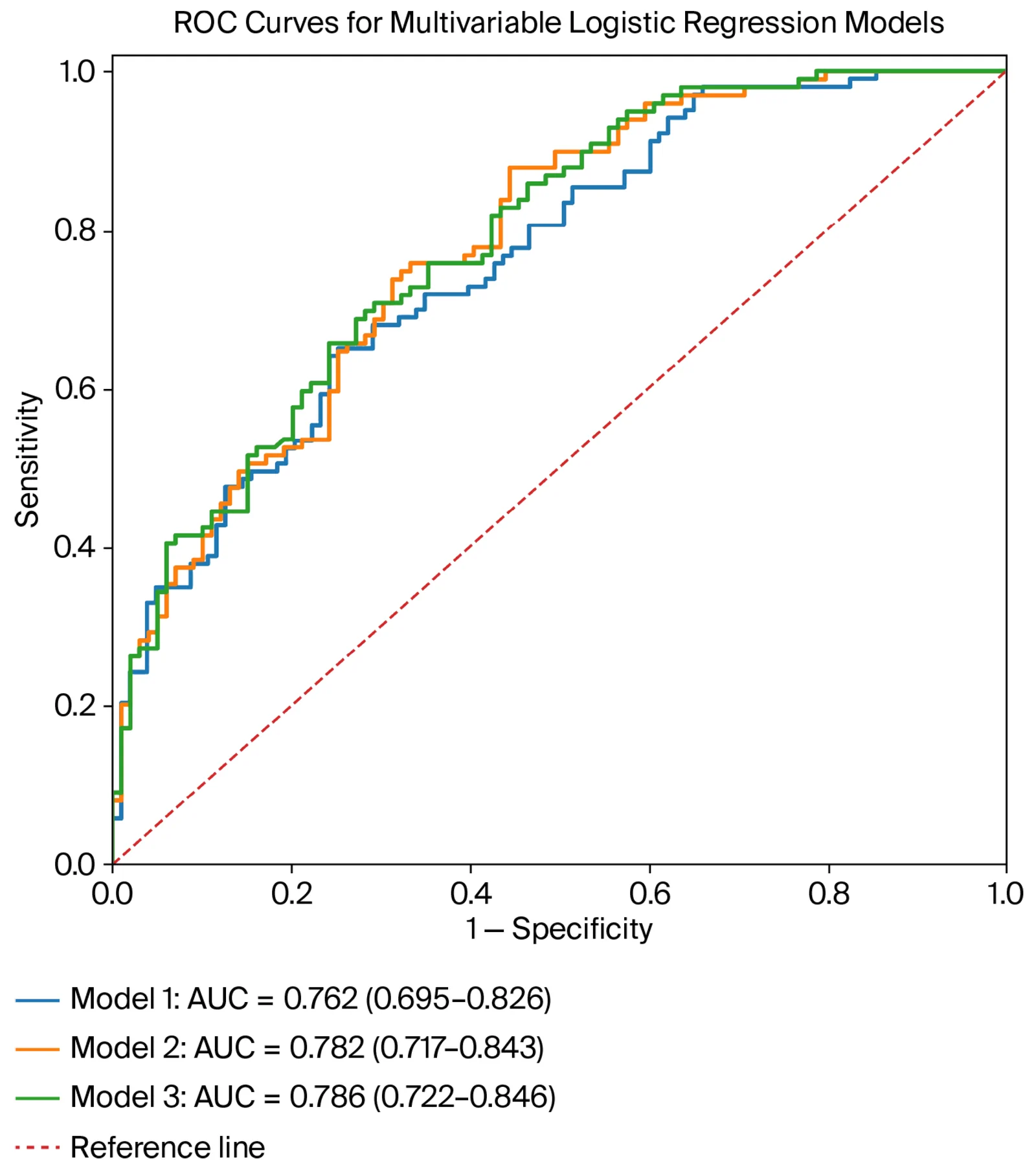
Receiver operating characteristic (ROC) curves comparing Models 1–3 for discriminating between participants with and without hypertension.

ROC curve analysis demonstrated acceptable discrimination for all three models (Figure 5). The AUCs were 0.76 (95% CI: 0.70–0.83), 0.78 (95% CI: 0.72–0.84), and 0.79 (95% CI: 0.72–0.85) for Models 1, 2, and 3, respectively (Table 6).

## 4. Discussion

The present study investigated the associations between body composition parameters and hypertension among Thai adults. The principal findings were that individuals with hypertension exhibited significantly greater adiposity-related body composition characteristics, including higher body weight, BMI, body fat percentage, fat mass, visceral fat level, and waist-to-hip ratio, than normotensive individuals. In Model 3, bioelectrical impedance analysis (BIA)-derived visceral fat level, but not skeletal muscle mass, was associated with hypertension after adjustment for age and sex. However, visceral fat level was not statistically significant when modeled together with body fat percentage in Model 2; therefore, the observed association should be interpreted as dependent on model specification. Moreover, the BIA-derived visceral fat level represents a surrogate estimate rather than a direct imaging-based measurement of visceral adipose tissue.

Participants with hypertension also reported a higher prevalence of current smoking and a lower prevalence of regular exercise than normotensive individuals. These findings are consistent with established evidence indicating that smoking and physical inactivity are important behavioral factors associated with elevated BP and cardiovascular risk [19]. Nevertheless, these variables were not included in the multivariable models because the present study focused specifically on body composition characteristics, and the relatively small number of current smokers in the study population may have reduced statistical stability.

The observed association between visceral fat accumulation and hypertension is consistent with current understanding of cardiometabolic pathophysiology. Unlike subcutaneous adipose tissue, visceral adipose tissue is highly metabolically active and exhibits increased secretion of pro-inflammatory cytokines, including tumor necrosis factor-α and interleukin-6, together with alterations in adipokine production [20,21]. Excess visceral fat promotes systemic inflammation, oxidative stress, endothelial dysfunction, insulin resistance, and activation of both the sympathetic nervous system and the renin–angiotensin–aldosterone system [22,23]. These mechanisms contribute to increased vascular resistance, impaired vascular compliance, sodium retention, and ultimately elevated BP [24,25]. These mechanisms provide biological plausibility for the observed association between visceral adiposity and hypertension and are consistent with recent epidemiological and systematic-review evidence [26,27,28,29,30,31,32].

Previous investigations have reported significant differences in body composition profiles between individuals with favorable and unfavorable cardiometabolic health status despite similar obesity classifications [33,34,35]. Although the association between visceral fat level and hypertension was statistically significant, its clinical significance should be interpreted with caution given the modest effect size and the cross-sectional design of the study.

In addition to overall adiposity measures, several regional fat distribution parameters differed between normotensive and hypertensive participants. Although abdominal fat mass tended to be higher among participants with hypertension, the between-group difference was not statistically significant. In contrast, fat accumulation in both upper and lower extremities was significantly greater in participants with hypertension, suggesting a generalized increase in adiposity accompanying greater visceral fat accumulation. In contrast, skeletal muscle mass and segmental muscle mass parameters were not independently associated with hypertension in the multivariable model. Although skeletal muscle plays an important role in metabolic regulation and insulin-mediated glucose disposal [36], the present findings suggest that absolute muscle mass and regional muscle mass may not be a primary determinant of hypertension after accounting for age, sex, and visceral adiposity. Previous studies have reported inconsistent associations between muscle mass and BP, possibly due to differences in study populations, body composition assessment methods, and adjustment strategies [37,38]. It is possible that muscle quality, muscle strength, physical performance, or intramuscular fat infiltration may be more closely related to cardiovascular health than muscle mass alone.

The present findings have important public health implications. Our results support recent recommendations advocating the incorporation of body fat distribution into cardiometabolic risk assessment [39,40], particularly among Asian populations, who tend to accumulate visceral adipose tissue at relatively lower BMI levels than Western populations [41,42]. Accordingly, assessment of visceral adiposity may facilitate the identification of individuals at increased cardiometabolic risk and support the implementation of targeted lifestyle interventions, including dietary modification, regular physical activity, and weight management strategies aimed at reducing central adiposity. Such approaches may contribute to both hypertension prevention and broader cardiometabolic risk reduction [43]. The discriminatory performance of the final model further suggests that body composition assessment, particularly visceral adiposity evaluation, may provide useful complementary information for identifying individuals with a higher likelihood of hypertension in community and clinical settings. Nevertheless, visceral adiposity should not be viewed as the sole determinant of hypertension. Hypertension is a multifactorial condition arising from complex interactions among genetic, biological, behavioral, environmental, and socioeconomic factors. Previous studies have shown that reproductive history and environmental factors may contribute to obesity and obesity-associated hypertension later in life, highlighting the complexity of the pathways involved in hypertension development [44]. Therefore, while the present findings support the importance of visceral adiposity in hypertension risk assessment, they should be interpreted within the broader context of the multifactorial mechanisms underlying hypertension.

Several limitations should be considered when interpreting the findings. First, the case–control design precludes causal inference and does not allow determination of temporal relationships between body composition parameters and hypertension. In addition, the case–control design involved intentional recruitment of comparable numbers of normotensive and hypertensive participants; therefore, the findings should not be interpreted as reflecting the population prevalence of hypertension or used for risk prediction at the population level. Second, body composition was assessed using BIA with the InBody 270. The visceral fat level provided by the device represents a surrogate estimate of visceral adiposity rather than a direct imaging-based measurement of visceral adipose tissue. Although BIA-derived indices have demonstrated utility for body composition assessment in both clinical and research settings, computed tomography (CT) and magnetic resonance imaging (MRI) remain the reference standards for quantifying visceral adipose tissue. Therefore, the possibility of measurement error and misclassification of visceral adiposity cannot be excluded, and the findings should be interpreted in light of this limitation. Third, residual confounding from unmeasured or imprecisely measured factors, including dietary habits, physical activity levels, medication use, smoking, alcohol consumption, and socioeconomic status, cannot be excluded. Fourth, lifestyle variables were not included in the main multivariable models because the primary objective was to evaluate body composition characteristics associated with hypertension. In addition, the low prevalence of smoking and alcohol consumption limited the stability of model estimates. Fifth, hypertension classification was based on BP measurements obtained during a single study visit together with self-reported physician diagnosis and treatment status; therefore, some degree of BP misclassification cannot be excluded. Finally, participants were recruited from a limited geographic region of Thailand, which may restrict the generalizability of the findings to other populations.

## 5. Conclusions

Higher BIA-derived visceral fat level was associated with hypertension among Thai adults after adjustment for age, sex, and skeletal muscle mass, whereas skeletal muscle mass showed no independent association within the same model. These findings suggest that visceral fat distribution may provide complementary information in cardiometabolic assessment. However, the case–control design, potential residual confounding, and use of a surrogate estimate of visceral adiposity preclude causal or population-level predictive interpretation. Prospective studies using imaging-based assessments are warranted to confirm these findings.

## Figures and Tables

**Table 1:** Demographic, clinical, anthropometric, body composition, and hemodynamic characteristics of normotensive and hypertensive participants.

Characteristic	Total (*n* = 208)	Normotension (*n* = 103)	Hypertension (*n* = 105)	Effect Size	*p*-Value
Demographic characteristics					
Age (years)	46.50 ± 17.94	40.95 ± 17.68	51.93 ± 16.55 *	0.64	<0.001
Sex, *n* (%)					
Female	161 (77.40)	88 (85.44)	73 (69.52) *	0.39	0.010
Male	47 (22.60)	15 (14.56)	32 (30.48) *	0.39	0.010
Clinical and lifestyle characteristics					
- Newly diagnosed hypertension, *n* (%)			59 (56.19)		-
- Treated hypertension, *n* (%)			46 (43.81)		-
Treatment status					
- Diuretics, *n* (%)		-	3 (6.52)		-
- ACE inhibitors, *n* (%)		-	9 (19.57)		-
- Angiotensin receptor blockers, *n* (%)		-	15 (32.61)		-
- Beta-blockers, *n* (%)		-	5 (10.87)		-
- Calcium channel blockers, *n* (%)		-	18 (39.13)		-
Lipid-lowering medications, *n* (%)		16 (15.53)	19 (18.10)	0.07	0.622
Current smoking, *n* (%)		1 (0.97)	7 (6.67) *	0.30	0.033
Alcohol consumption, *n* (%)		14 (13.59)	18 (17.14)	0.10	0.478
Regular exercise, *n* (%)		54 (52.43)	36 (34.29) *	0.37	0.008
Anthropometric and body composition characteristics					
Weight (kg)	64.57 ± 15.19	61.21 ± 12.91	67.94 ± 16.56 *	0.45	0.001
Height (m)	1.60 ± 0.08	1.60 ± 0.07	1.60 ± 0.09	0.11	0.437
BMI (kg/m^2^)	25.12 ± 5.09	23.96 ± 4.37	26.27 ± 5.49 *	0.47	0.001
Body fat (%)	33.28 ± 7.89	32.01 ± 7.95	34.56 ± 7.66 *	0.33	0.023
Fat mass (kg)	22.11 ± 9.15	19.59 ± 7.35	24.89 ± 10.14 *	0.60	<0.001
Water mass (kg)	30.32 ± 7.49	29.99 ± 6.38	30.65 ± 8.48	0.15	0.540
Skeletal muscle mass (kg)	23.31 ± 5.62	22.43 ± 5.09	24.19 ± 6.00 *	0.32	0.027
Protein mass (kg)	8.35 ± 2.00	8.05 ± 1.79	8.68 ± 2.18 *	0.32	0.047
Mineral mass (kg)	2.96 ± 0.67	2.86 ± 0.57	3.07 ± 0.76 *	0.32	0.044
Visceral fat level	9.70 ± 4.23	8.57 ± 3.84	10.83 ± 4.31 *	0.55	<0.001
Waist-to-hip ratio	0.87 ± 0.06	0.86 ± 0.05	0.88 ± 0.07 *	0.34	0.037
BMR (kcal)	1292.68 ± 218.68	1261.05 ± 186.03	1324.31 ± 243.93 *	0.29	0.042
Fitness score	67.78 ± 6.28	69.05 ± 5.37	66.37 ± 6.93 *	0.44	0.007
Hemodynamic characteristics					
SBP (mmHg)	124.27 ± 20.22	110.14 ± 10.71	138.27 ± 17.50 *	1.94	<0.001
DBP (mmHg)	77.72 ± 10.93	71.05 ± 6.39	84.33 ± 10.49 *	1.53	<0.001
HR (beats/min)	76.69 ± 10.65	76.21 ± 9.15	77.16 ± 11.97	0.09	0.524
PP (mmHg)	46.55 ± 13.90	39.08 ± 8.61	53.94 ± 14.21 *	1.26	<0.001
MAP (mmHg)	92.79 ± 14.63	84.08 ± 7.00	101.34 ± 15.13 *	1.45	<0.001
RPP × 10^−2^ (mmHg·beats/min)	95.53 ± 22.11	84.09 ± 14.03	106.86 ± 22.85 *	1.20	<0.001
SpO_2_ (%)	98.21 ± 1.02	98.24 ± 1.02	98.18 ± 1.02	0.06	0.673

Data are presented as mean ± standard deviation (SD) or *n* (%), as appropriate. Categorical variables were compared using the χ^2^ test. Continuous variables were compared using the independent samples *t*-test or Mann–Whitney U test, as appropriate. Effect sizes are presented as Cohen’s *d* for continuous variables and Cohen’s *h* for categorical variables. ACE, angiotensin-converting enzyme; BMI, body mass index; BMR, basal metabolic rate; DBP, diastolic blood pressure; HR, heart rate; MAP, mean arterial pressure; PP, pulse pressure; RPP, rate-pressure product; SBP, systolic blood pressure; SpO_2_, peripheral oxygen saturation. * *p* < 0.05 vs. normotension. Regular exercise: ≥150 min/week of moderate-intensity physical activity or equivalent.

**Table 2:** Segmental muscle and fat mass in normotensive and hypertensive participants.

Segment	Total (*n* = 208)	Normotension (*n* = 103)	Hypertension (*n* = 105)	Effect Size	*p*-Value
Left arm muscle (kg)	2.04 ± 0.73	1.93 ± 0.67	2.16 ± 0.78	0.33	0.043
Right arm muscle (kg)	2.09 ± 0.73	1.97 ± 0.68	2.22 ± 0.77	0.35	0.028
Left leg muscle (kg)	6.57 ± 1.76	6.28 ± 1.52	6.85 ± 1.92	0.33	0.041
Right leg muscle (kg)	6.59 ± 1.77	6.33 ± 1.54	6.88 ± 1.96	0.31	0.052
Abdominal muscle (kg)	18.57 ± 4.67	17.95 ± 4.11	19.27 ± 5.16	0.29	0.076
Left arm fat (kg)	1.61 ± 0.96	1.37 ± 0.67	1.88 ± 1.15	0.54	0.001
Right arm fat (kg)	1.63 ± 0.99	1.38 ± 0.69	1.91 ± 1.18	0.55	0.001
Left leg fat (kg)	3.45 ± 1.47	3.07 ± 1.10	3.88 ± 1.69	0.58	<0.001
Right leg fat (kg)	3.44 ± 1.45	3.11 ± 1.08	3.80 ± 1.72	0.49	0.003
Abdominal fat (kg)	11.93 ± 11.56	10.74 ± 11.82	13.27 ± 11.17	0.22	0.164

Data are presented as mean ± standard deviation (SD). Continuous variables compared by independent *t*-test or Mann–Whitney U test. Cohen’s *d* was applied to demonstrate effect size.

**Table 3:** Univariate logistic regression analysis of demographic and body composition factors associated with hypertension.

Variable	OR	95% CI	*p*-Value
Age (years)	1.04	1.02–1.05	<0.001
Sex	2.57	1.29–5.11	0.007
BMI (kg/m^2^)	1.10	1.04–1.17	0.002
Body fat (%)	1.04	1.01–1.08	0.025
Fat mass (kg)	1.08	1.03–1.12	0.001
Visceral fat level	1.15	1.07–1.23	<0.001
Skeletal muscle mass (kg)	1.06	1.01–1.12	0.030

Total *n* = 208. BMI, body mass index; ORs represent the odds of hypertension associated with a one-unit increase in each continuous variable, 95% confidence intervals (CIs), and *p*-values were estimated using univariate logistic regression models with hypertension as the dependent variable. Sex was coded as female = 0 and male = 1.

**Table 4:** Multivariable logistic regression models of independent factors associated with hypertension.

Variable	Model 1 Adjusted OR (95% CI)	*p*-Value	Model 2 Adjusted OR (95% CI)	*p*-Value	Model 3 Adjusted OR (95% CI)	*p*-Value
Age (years)	1.06 (1.04–1.08)	<0.001	1.06 (1.04–1.08)	<0.001	1.06 (1.04–1.09)	<0.001
Male sex	5.95 (2.44–14.51)	<0.001	6.90 (2.21–21.55)	0.001	5.35 (1.69–16.95)	0.004
BMI (kg/m^2^)	1.11 (1.04–1.19)	0.002	-	-	-	-
Body fat (%)	-	-	1.00 (0.91–1.09)	0.950	-	-
Visceral fat level	-	-	1.17 (1.00–1.37)	0.055	1.16 (1.06–1.26)	<0.001
Skeletal muscle mass (kg)	-	-	-	-	1.03 (0.95–1.12)	0.438

Total *n* = 208. BMI, body mass index; odds ratios (ORs), 95% confidence intervals (CIs), and *p*-values were estimated using multivariable logistic regression models with hypertension as the dependent variable. Model 1: Age + Sex + BMI; Model 2: Age + Sex + Body fat (%) + Visceral fat level; Model 3: Age + Sex + Skeletal muscle mass + Visceral fat level. Sex was coded as female = 0 and male = 1.

**Table 5:** Goodness-of-fit and discrimination statistics for the multivariable logistic regression models.

Model	AUC	Hosmer–Lemeshow *p*-Value	Nagelkerke R^2^	Max VIF
Model 1	0.76	0.62	0.28	1.32
Model 2	0.78	0.71	0.36	2.11
Model 3	0.79	0.75	0.37	1.58

Total *n* = 208. AUC, area under the ROC curve; VIF, variance inflation factor. Higher AUC values indicate better discrimination, whereas lower VIF values indicate lower multicollinearity.

**Table 6:** Receiver operating characteristic (ROC) analysis of the multivariable logistic regression models.

Model	Predictors	AUC	95% CI
Model 1	Age + Sex + BMI	0.76	0.70–0.83
Model 2	Age + Sex + Body fat (%) + Visceral fat level	0.78	0.72–0.84
Model 3	Age + Sex + Skeletal muscle mass + Visceral fat level	0.79	0.72–0.85

Total *n* = 208. BMI, body mass index. The 95% confidence intervals were estimated for the area under the ROC curve (AUC).

## Data Availability

The data are available upon request from the corresponding author. Restrictions apply to the availability of these data due to privacy and ethical considerations.

## Abbreviations

The following abbreviations are used in this manuscript:

ACE	Angiotensin-converting enzyme
AUC	Area under the receiver operating characteristic curve
BIA	Bioelectrical impedance analysis
BMI	Body mass index
BMR	Basal metabolic rate
BP	Blood pressure
CI	Confidence interval
CT	Computed tomography
DBP	Diastolic blood pressure
HR	Heart rate
HTN	Hypertension
MAP	Mean arterial pressure
MRI	Magnetic resonance imaging
NTN	Normotension
OR	Odds ratio
PP	Pulse pressure
ROC	Receiver operating characteristic
RPP	Rate-pressure product
SBP	Systolic blood pressure
SD	Standard deviation
SpO_2_	Peripheral oxygen saturation
VIF	Variance inflation factor
